# Mohs micrographic surgery in rare cutaneous tumors: a retrospective study at a Brazilian tertiary university hospital^⋆^

**DOI:** 10.1016/j.abd.2022.01.009

**Published:** 2022-11-08

**Authors:** Thais Helena Buffo, Rafael Fantelli Stelini, Juliana Yumi Massuda Serrano, Luciana Takata Pontes, Renata Ferreira Magalhães, Aparecida Machado de Moraes

**Affiliations:** a Discipline of Dermatology, Faculty of Medical Sciences, Universidade Estadual de Campinas, Campinas, SP, Brazil; b Department of Pathological Anatomy, Faculty of Medical Sciences, Universidade Estadual de Campinas, Campinas, SP, Brazil

**Keywords:** Acrospiroma, Adenocarcinoma, Carcinoma, Merkel cell, Dermatofibrosarcoma, Mohs surgery, Skin neoplasms

## Abstract

**Background:**

Mohs micrographic surgery is an established technique in the treatment of cutaneous neoplasms. It offers higher cure rates and the main indications are non-melanoma malignant skin tumors. Few studies have been performed on the treatment of rare tumors through this technique.

**Objective:**

To study rare skin tumors and rare variants of basal cell carcinoma and squamous cell carcinoma submitted to Mohs micrographic surgery in a tertiary service in relation to frequency, disease-free evolution, and applicability of this surgical procedure for this group of tumors.

**Methods:**

This was a retrospective observational study including rare skin tumors and less common variants of basal cell carcinoma and squamous cell carcinoma treated using Mohs micrographic surgery, between October 2008 and April 2021.

**Results:**

During the study period, 437 tumors were treated using Mohs micrographic surgery, and 22 (5%) rare skin tumors were selected. The tumors comprised three dermatofibrosarcomas protuberans, two atypical fibroxanthomas, two spiradenomas, two hypercellular fibrohistiocytomas, one primary cutaneous adenocarcinoma, one trichoblastoma, one porocarcinoma, one chondroid syringoma, one cutaneous angiosarcoma, one Merkel cell carcinoma, and one sebaceous carcinoma. Six other cases of rare basal cell carcinoma variants with trichoepitheliomatous differentiation, metatypical basal cell carcinoma, and clear cell squamous cell carcinoma were included. There were no cases of recurrence after an average of six years of follow-up.

**Study limitations:**

This is a retrospective study on rare neoplasms carried out in a single referral center, and this surgical technique isn’t widely available in the public service.

**Conclusion:**

This retrospective case series showed that Mohs micrographic surgery is an appropriate treatment for rare skin tumors. They corresponded to 5% of the tumors treated by the technique during a 12-year-period, with no recurrences identified.

## Introduction

Skin cancer is the most frequent in Brazil and accounts for about 30% of all recorded malignant tumors.^1^ It has high cure rates if detected early but can result in significant morbidity, if not correctly diagnosed and treated, especially in tumors with more aggressive and infiltrative histopathological types.^2^, ^3^

Mohs micrographic surgery (MMS) is currently considered the most effective method for treating many types of skin cancer.^4^ It is characterized by lesion identification, accurate mapping of all process stages, and complete evaluation of tumor margins. The relationship between the presence of a neoplasm on histopathology and its correct location on the surgical map is essential for complete lesion resection and healthy tissue preservation.^4^ It offers the highest cure rates for many of the most frequent cutaneous neoplasms, such as basal cell carcinoma (BCC) and squamous cell carcinoma (SCC) in their classic forms also in rare mixed forms, including BCC with adnexal differentiation, squamous or metatypical BCC or clear cell SCC. There are also other uncommon skin tumors, with high risk of recurrence, for which there are no well-established criteria regarding treatment and that may benefit from this technique.^5^, ^6^, ^7^

Rare or uncommon skin tumors do not have an exact definition and in this group, benign or malignant skin neoplasms of low prevalence can be included, which, in addition to being rare, can also recur. Because of the lack of uniformity regarding the reported cases, the true incidence is unknown.^7^ They can be divided, according to their histological origin, into some groups, with some main tumors. Briefly, they can be grouped as shown in Table 1.^7^, ^8^, ^9^, ^10^, ^11^, ^12^, ^13^

**Table 1:** Rare cutaneous neoplasms divided according to histological origin and examples of each group.^7^, ^8^, ^9^, ^10^, ^11^, ^12^, ^13^.

Rare cutaneous neoplasms		
Adnexal	Follicular differentiation	Tricoblastoma
		Trichoepithelioma
		Trichilemmal carcinoma
	Sebaceous differentiation	Sebaceous carcinoma
	Sudoriparous differentiation	Spiradenoma
		Hidradenoma
Vascular	Angiosarcoma, Kaposi sarcoma	
Neural and neuroendocrine	Merkel cell carcinoma	
Fibrohistiocytic	Dermatofibroma; atypical and cellular variants	
	Atypical fibroxanthoma	
	Dermatofibrosarcoma protuberans	
Muscular, adipocytic and cartilaginous	Leiomyosarcoma, chondroma	

Those of adnexal origin may show follicular differentiation, such as trichilemmal carcinoma and trichoblastoma; sebaceous differentiation, such as sebaceous carcinoma; or sudoriparous differentiation, such as microcystic adnexal carcinoma (MAC) and spiradenoma. MAC is an aggressive malignant neoplasm originating from the sweat glands, with dense stroma surrounding the tumor islands and extensive local infiltration.^14^ There are also those of vascular origin, such as angiosarcoma.^10^

One example of a tumor of neuroendocrine origin is Merkel cell carcinoma (MCC), an aggressive neuroendocrine skin tumor with increasing prevalence in recent years, reaching 0.7 cases per 100,000 inhabitants in the USA. It has a high rate of metastasis and mortality, with a 5-year survival of 62% in the early stages and 13.5% in the advanced stages.^11^, ^15^, ^16^, ^17^

Also recognized is the group of neoplasms of fibrohistiocytic origin, comprising neoplasms such as dermatofibrosarcoma protuberans (DFSP) and atypical fibroxanthoma (AFX).^12^ DFSP is a slow-growing, asymmetrical malignant tumor with an poorly defined subclinical extent. Histopathologically, there is diffuse infiltration of spindle cells, with a storiform pattern. DFSP has reported incidence rates ranging from 0.8 to 5 per million inhabitants.^18^ A population study from 1972 to 2002 showed 4.2 per million.^19^ AFX is a fibrohistiocytic tumor of intermediate malignant potential that accounts for about 0.002% of skin tumors, according to a ten-year study from New Zealand.^20^

There are also tumors of muscular, adipocytic, or cartilaginous origin.^13^ Many of these tumors do not have a prevalence and incidence estimate due to the small number of cases, with studies based only on small reports.

The main goal of treatment is an oncological cure, but one should also aim to preserve function and maximum healthy tissue in addition to a satisfactory cosmetic result.^21^

In relation to less prevalent skin tumors, non-surgical methods are rarely indicated. Radiotherapy and systemic treatments involving immunotherapy may be alternatives in patients with contraindications for the surgical procedure.^22^ Moreover, in the case of rare skin tumors with a recurrent characteristic, surgical methods in which it is not possible to assess the surgical margins, such as electrocoagulation and cryosurgery, are not routinely indicated.^21^, ^23^

The most frequently used method is conventional surgery with wide margins, characterized by tumor excision and surgical wound closure. The visible lesion is removed with a standardized and variable safety margin, according to the tumor type and characteristics. The standardization of safety margins is established for BCC and SCC, but it is not well established for less prevalent tumors.^5^, ^6^

The surgical specimen is sent for anatomopathological evaluation. The pathologist confirms the histopathological subtype and the involvement or not of the margins in the assessed sections. Multiple sequential cuts are performed on the specimen; however, it is not possible to assess100% of the margins, which is done by sampling. For this reason, tumor escape may occur at an unassessed margin, resulting in a false negative result.^24^

Mohs micrographic surgery is the best-known method among the options of Micrographic Surgery with Total Intraoperative Margin Control (MSTIOMC). All margins are assessed during surgery, with frozen sections and reconstruction during a single surgical time, unlike conventional surgery and simple freezing, which is also performed by sampling.^4^, ^5^, ^6^, ^25^

MSTIOMC is the most effective technique in the treatment of cutaneous neoplasms. The removal of the clinically delimited tumor mass is performed with a minimum safety margin, which varies according to the histopathological tumor type. A thorough mapping of all margins of the obtained fragment is necessary, allowing a perfect correlation with the surgical defect left in the patient. Markings are made on the specimen, reference points, and orientation between the tumor and the skin, in addition to the fragment staining with different colors. The colors are important for the identification and orientation of the studied margins, in addition to the spatial location of an eventual tumor found in the fragments. The mapping can be made on paper or digitally, through photos and editing applications.^4^, ^25^

The samples are submitted to freezing in the cryostat and the slides are stained with hematoxylin & eosin (H&E) and viewed under an optical microscope for evaluation by the micrographic surgeon. Once the presence of neoplasm is detected in any of the fragments, the affected area is identified on the map, so that a targeted resection of the additional fragment can be performed, sparing healthy tissue. The tumor excision ends when all margins are negative for neoplasia.^4^, ^25^

Among the different methods of MSTIOMC, there are the classic MMS at 45 degrees, the variant MMS at 90 degrees, used in the present study, and the Munich Technique, all of which have similar cure rates.^25^ The choice of method will depend on the training and experience of the professional, without consequences for the patient who will undergo the procedure.

MMS, despite being widely recognized for the treatment of several of these tumors, both common and rare, with superior cure rates, remains underused. Ideally, it could be considered the first choice in high-risk, primary, or recurrent tumors.^26^, ^27^ However, due to reduced availability, higher cost of implantation and maintenance, and the need for specialized professionals that have been trained in the method and cutaneous oncology during a long training process, it is mainly indicated in recurrent tumors, after several previous treatments, which reduces its effectiveness.

Most studies published in MMS have as their topic the treatment of BCC and SCC, the most common types in this group and which have well-established indication criteria. There is, however, indication for surgical treatment for multiple less common tumors.^5^, ^6^, ^26^, ^27^, ^28^, ^29^, ^30^, ^31^, ^32^, ^33^, ^34^, ^35^, ^36^

Rare skin tumors are commonly treated by the conventional surgical technique with wide margins, and there is no consensus regarding the standardization of these margins. Nevertheless, incomplete excisions and recurrence occur often.^5^, ^6^, ^37^, ^38^, ^39^

Flohil et al., in 2017,^5^ reported a study of rare tumors treated using MMS at a university center in the Netherlands. The retrospective review included 80 rare tumors, consisting of dermatofibrosarcoma protuberans (33%), atypical fibroxanthoma (27.5%), Merkel cell carcinoma (11.25%), microcystic adnexal carcinoma (10%), sebaceous carcinoma (7.5%), extramammary Paget's disease (2.5%), spiradenocarcinoma (1.25%), among others. It concluded that MMS is an appropriate treatment for the rare tumors with only two cases of recurrence, both atypical fibroxanthomas.^5^

DFSP is the rare tumor with the highest number of reports of treatment using MMS.^26^, ^29^ The subclinical spread of this neoplasm makes its complete removal difficult, with high recurrence rates in case series. Cernea et al.^26^ justify the indication of the MMS technique for the treatment of DFSP in the guidelines of the Brazilian Society of Dermatology, based on the available evidence that indicates a lower rate of recurrence. Although it is the rare tumor with the highest number of reports, there is a lack of better quality, randomized and controlled studies with longer follow-ups.

This tumor has high recurrence rates, reaching 26% to 53% in some case series.^18^, ^19^, ^29^ A systematic review gathered data on DFSP treated by MMS from 1995 to 2011, including 23 non-randomized studies, of which four were comparative ones. The observed recurrence rate was 1.1% in the MMS group, *versus* 6.3% in the conventional surgery group.^29^

Although routinely indicated for malignant tumors, MMS can also be used in cases of large, poorly defined benign tumors, with a high risk of recurrence. One example is trichoblastoma, a rare, slow-growing, well-circumscribed adnexal tumor. Even though it is a benign tumor, reports have shown trichoblastomas with a more aggressive course.^30^, ^39^ Although rare, the risk of malignant transformation exists and should be taken into account when indicating treatment and evaluating MMS.^30^

No Brazilian studies have been found that assessed several histopathological types of less frequent skin tumors, only individual case reports or case series, mainly of DFSP, the rare tumor most frequently treatded with MMS.^26^, ^30^

Reports involving the use of MMS in the treatment of less common skin tumors are scarce, but they report lower rates of recurrence,^5^, ^6^, ^26^, ^27^, ^28^, ^29^, ^30^, ^31^, ^32^, ^33^, ^34^, ^35^, ^36^ requiring further studies in this area.

## Patients and methods

### Study design

This was a retrospective observational study, including all cases of rare skin tumors or less common variants of BCC and SCC treated using the 90-degree variant Mohs micrographic surgery technique, carried out at the Division of Dermatology, Hospital de Clínicas, Unicamp, between October 2008 and April 2021.

### Research ethics committee

The study was approved by the Research Ethics Committee of the institution under counsel number 3,113,179, including the Free and Informed Consent Term (FICT). The informed consent was signed by the patients at the time of their outpatient follow-up appointment.

### Data collection

Data were collected by reviewing the medical files and photographic records of the procedures including a specific database of micrographic surgery at the service. All patients were over 18 years of age and came from the Hospital Skin Tumor Outpatient Clinic. After the procedure, the participants were seen at periodic clinical follow-ups, making it possible to assess tumor recurrence.

All treated patients were identified and included, at the time of the surgery, in a single registry, which allowed the accuracy of the collected data. In this registry, patient data such as name, hospital registration number, date of birth, sex, age at the time of surgery, and date of procedure were included. Neoplasm data, such as histopathological type, whether recurrent or primary, were also recorded, in addition to details of the procedure, such as number of stages, number of fragments, reconstruction, surgical map with photographic documentation, and clinical evolution.

### Inclusion criteria

All patients over 18 years of age, with a confirmed histopathological diagnosis of rare skin neoplasia or less prevalent variants of BCC and SCC, submitted to surgical treatment using Mohs micrographic surgery from 10/2008 to 04/2021 were included. The less prevalent variants of BCC and SCC that could be included were: BCC with adnexal or trichoepitheliomatous differentiation, metatypical BCC or basosquamous carcinoma, clear cell SCC and acantholytic SCC. The procedure was performed by dermatologists specialized in micrographic surgery, certified by the Brazilian Society of Dermatology, and followed the criteria established by the Research Ethics Committee.

### Histopathological examination and surgical technique

All patients were treated employing the 90-degree variant Mohs micrographic surgery technique, characterized by the separation of the fragment into lateral and deep margins with a 90-degree section, followed by cryostat freezing and 5-micron thick sections. The prepared slides were stained with hematoxylin-eosin and then evaluated by micrographic surgeons.^4^, ^25^ As these were rare tumors, the slides were also evaluated by a dermatopathologist. After selecting the patients, the slides prepared during the respective procedures were retrieved for photographic recording of the research.

### Analysis of results

A descriptive analysis of the rare tumors was carried out, as well as the description of the technique according to the number of stages, disease-free evolution, and cases of recurrence. Only the descriptive statistical analysis was performed, using the SPSS Statistics 20 software for Windows.

## Results

During the period from October 2008 to April 2021, 437 tumors were operated on using the Mohs micrographic surgery technique and 22 (5%) cases were selected because they were considered rare tumors or rare BCC and SCC variants. The other 95% corresponded to common subtypes of BCC and SCC, in addition to a case of lentigo maligna.

Regarding gender, 12 (54.5%) of the cases were female and ten (45.5%) were male. Regarding the location of the tumors, 15 (68.2%) were located on the head and neck, four (18.2%) on the trunk, and three (13.6%) on the extremities.

In total, 22 patients with a rare skin tumor were fully treated with MMS. The tumors were three dermatofibrosarcomas protuberans, two atypical fibroxanthomas, two spiradenomas, two atypical hypercellular fibrohistiocytomas, one primary cutaneous adenocarcinoma, one trichoblastoma, one porocarcinoma, one chondroid syringoma, one cutaneous angiosarcoma, one Merkel cell carcinoma, and one sebaceous carcinoma. Six other cases of rare variants of basal cell carcinoma and squamous cell carcinoma included four BCC with trichoepitheliomatous follicular differentiation, one metatypical BCC, and one clear-cell SCC.

The mean age of patients at the time of the MMS was 54 years. The youngest patients were 25 years old and the oldest was 84 years old. Seven patients were younger than 50 years (three cases of dermatofibrosarcoma protuberans, two hypercellular fibrohistiocytomas, one adenocarcinoma, and one atypical fibroxanthoma).

There were no cases of recurrence during the mean follow-up of 72 months. No case was excluded due to the minimum follow-up time, justifying cases with different clinical follow-up times, as described in Table 2. As all patients maintained their clinical follow-ups, it was possible for all of them to be evaluated by a dermatologist at the time of data collection to identify possible tumor recurrence.

**Table 2:** Characteristics of the patients, tumors and the surgical procedure.

Patients and tumor characteristics	Total	DFSP	AFX	SP	HF	AdeCa	TRB	PoCa	CS	ANS	MCC	SC	Others^a^
**Total number of tumors**	22	3	2	2	2	1	1	1	1	1	1	1	6
Sex													
Male	10 (45.5%)	0	2	0	1	0	0	1	1	1	0	1	3
Female	12 (54.5%)	3	0	2	1	1	1	0	0	0	1	0	3
**Mean age at MMS**	54.7	35.3	64.5	57	27	29	66	71	62	73	50	79	61.77
**Number of stages**													
1	13 (59.1%)	2	2	1	2	1	0	1	0	0	1	0	3
2	8 (36.4%)	1	0	0	0	0	1	0	1	1	0	1	3
3	1 (4.5%)	0	0	1	0	0	0	0	0	0	0	0	0
**Mean number of fragments**	6.9	9	7.5	11	4	5	8	4	6	9	5	6	6.17
**Mean follow-up in months**	72.24	84.7	58.5	60.5	102	98	120	41	32	27	17	6	81
**Recurrence**	0	0	0	0	0	0	0	0	0	0	0	0	0
**Tumor location**													
Head and neck	15 (68.2%)	0	1	1	1	1	1	1	1	1	0	1	6
Trunk	4 (18.2%)	3	1	0	0	0	0	0	0	0	0	0	0
Extremities	3 (13.6%)	0	0	1	1	0	0	0	0	0	1	0	0
**Previous treatment**													
None	9 (40.9%)	0	0	0	1	0	0	1	1	1	1	1	3
Incomplete excision	13 (59.1%)	3	2	2	1	1	1	0	0	0	0	0	3
**Reconstruction**													
Complex	14 (63.6%)	0	1	1	1	0	1	1	1	1	1	1	5
Non-complex	8 (36.4%)	3	1	1	1	1	0	0	0	0	0	0	1

DFSP, Dermatofibrosarcoma protuberans; AFX, Atypical fibroxanthoma; SP, Spiradenoma; HF, Hypercellular fibrohistiocytoma; AdeCa, Adenocarcinoma; TRB, Trichoblastoma; PoCa, Porocarcinoma; CS, Chondroid syringoma; ANS, Angiosarcoma; MCC, Merkel cell carcinoma; SC, Sebaceous carcinoma.

a Others include rare variants of basal cell and squamous cell carcinoma.

The three cases with the shortest follow-up time comprised a case of cutaneous angiosarcoma (27 months), a Merkel cell carcinoma (17 months), and a sebaceous carcinoma (6 months) but all of them are under strict clinical follow-up for recurrences and distant disease and were included aiming at emphasizing the applicability of the surgical technique for this group of tumors.

Among the selected cases, 13 (59.1%) of them had already been submitted to previous conventional surgery; however, due to incomplete excision margins, they were indicated for treatment with MMS.

**Figure 1:**
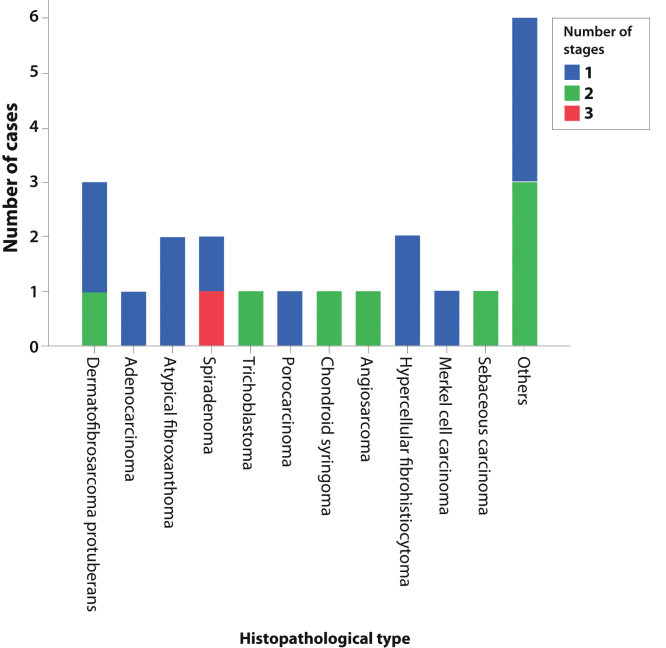
Number of stages required for complete tumor excision according to the diagnosis or treated lesion.

Fig. 1 shows the required number of MMS stages for complete tumor excision. The only case that required three stages was a recurrent spiradenoma.

In 14 (63.6%) cases, the patients required complex reconstruction after the procedure, involving grafts or flaps, and in 8 (36.4%) there was the possibility of non-complex reconstruction, by primary closure or by second intention. Table 2 includes a description of the 22 treated cases, summarizing the data.

## Discussion

This retrospective case series shows that MMS is an appropriate treatment for rare skin tumors. In contrast with BCC and SCC, which have clear indications for MMS performance, by classifying lesions into high and low risk, less prevalent neoplasms still do not have well-established criteria. It may be indicated for primary or recurrent, poorly defined lesions located in anatomical regions with the need to spare healthy tissue or with a high recurrence characteristic, such as DFSP.^5^, ^6^ In relation to rare benign skin tumors, it would be indicated in recurrent, large, poorly delimited lesions, or those at risk of malignant transformation.^5^, ^6^

With the benefit of a full assessment of tumor margins, MMS provides lower recurrence rates also for this group of tumors but each case needs to be evaluated individually.^5^, ^6^, ^7^, ^8^, ^26^, ^27^, ^28^, ^29^, ^30^, ^31^, ^32^, ^33^, ^34^, ^35^, ^36^

Less prevalent cutaneous neoplasms corresponded to approximately 5% of all tumors treated at the service using the MMS technique during the 12-year period. There were no cases of recurrence, with an average of six years of follow-up, and the majority of cases (59.1%) were tumors with previous incomplete excision and, therefore, a higher risk of recurrence.

It was also observed that, despite not having been selected because it is not a rare skin tumor, the case of lentigo maligna treated with MMS with intraoperative immunohistochemistry reinforces the possibilities for expansion of the indications of the technique.^34^ Immunohistochemistry, although not widely available, with a high cost and increased surgical time, can be of benefit in the evaluation of many of these tumors during MMS.^34^

There is some difficulty in defining rare tumors and determining incidence and prevalence data, due to the small number of cases regarding some of them. Several other tumors for which there have already been reports of treatment with MMS were not included in this series, such as microcystic adnexal carcinoma and extramammary Paget's disease.

Although the study was carried out in a tertiary hospital, a referral center for cutaneous oncology and high-complexity surgery, with high demand, the performance of MMS is limited by the availability of the technique.

It has also been observed that it is difficult to carry out prospective and randomized studies in a single research center, involving less frequent neoplasms.^5^, ^6^

There were no cases of local or distant recurrence in this study, after an average of six years of follow-up, which is in agreement with other data in the literature.^5^, ^6^ A university center in the Netherlands reported a retrospective case series of rare tumors treated with MMS. A total of 80 tumors were included and it was concluded that MMC is an appropriate treatment for this group of tumors, with only two cases of recurrence.^5^

Regarding the location of the lesions in this series, 68.2% of the tumors were located in the head and neck, which can be explained by the MMS indication criteria and not by the higher prevalence of these tumors in this location. This is a region with a higher risk for recurrence and where there is also the need to spare healthy tissue. In this anatomical topography, the safety of a complete evaluation of the tumor margins prior to reconstruction of the surgical defect is also one of the criteria for choosing the method. An exception is DFSP, a neoplasm with the highest prevalence on the trunk and limbs.^18^, ^19^

DFSP is the rare tumor with the highest number of studies with MMS.^26^, ^29^, ^32^ In 58 cases of DFSP, the extent of tumor invasion was calculated by studying the margins using frozen sections used in MMS and it was observed that 15.5% of tumors would have been incompletely excised if the 3 cm surgical margin had been used.^35^ A retrospective Canadian study of 48 cases of primary DFSP, 28 treated by conventional surgery and 20 by MMS, found 21.4% of positive margins in the first group.^32^

**Figure 2:**
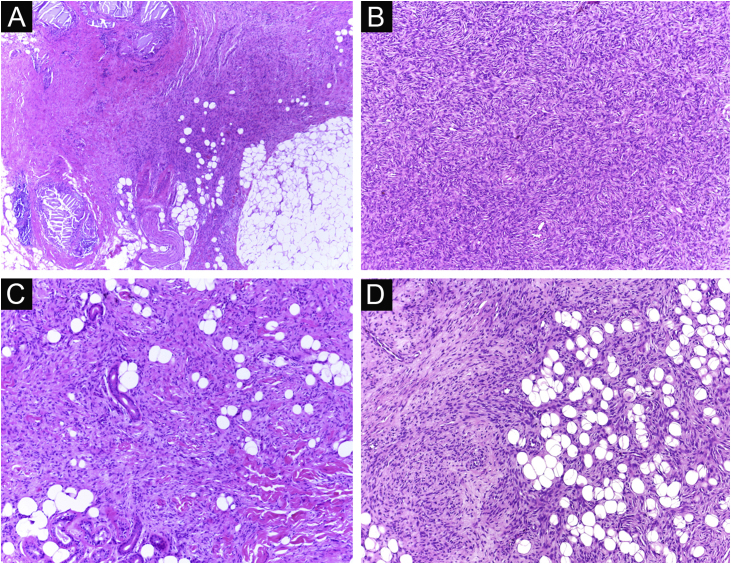
Dermatofibrosarcoma protuberans (Paraffin-embeded section, Hematoxylin & eosin). (A) (top left image): recurrent neoplasm, invading the hypodermis on the right side of the image and, on the left, an area of cicatricial fibrosis and granulomatous foreign body reaction secondary to previous conventional surgery. (B) (top right image): dense area of storiform pattern. (C and D) (bottom right and left images): infiltrative periphery of the neoplasm, with fascicles of monomorphic spindle cells, invading fibroconnective tissue on the left, and adipose tissue in both. Source: Archives of Dermatology/Pathological Anatomy HC-Unicamp.

It has been reported that DFSP is associated with more stages and large defects, as there is no consensus regarding the initial margin, which is greater compared to other tumors, often starting at one cm. Even so, it is a smaller margin than the three cm margin frequently used in conventional surgery. This study showed that three cases were treated with MMS, incompletely excised in the first approach, two of them with negative margins in only one stage. Both cases were submitted to MMS soon after the identification of compromised margins in the conventional surgery; however, they still presented residual tumor. The rapid indication of the procedure may justify obtaining free margins in the first stage, with a less subclinical extension of the residual lesion. The two cases have 138 and 110 months of follow-up each, with no signs of recurrence. The long follow-up time without evidence of clinical disease reinforces the appropriate indication of the surgical technique. Fig. 2 depicts the histopathological characteristics of DFSP, with deep and asymmetrical tumor infiltration, which corroborates the indication of MMC.

**Figure 3:**
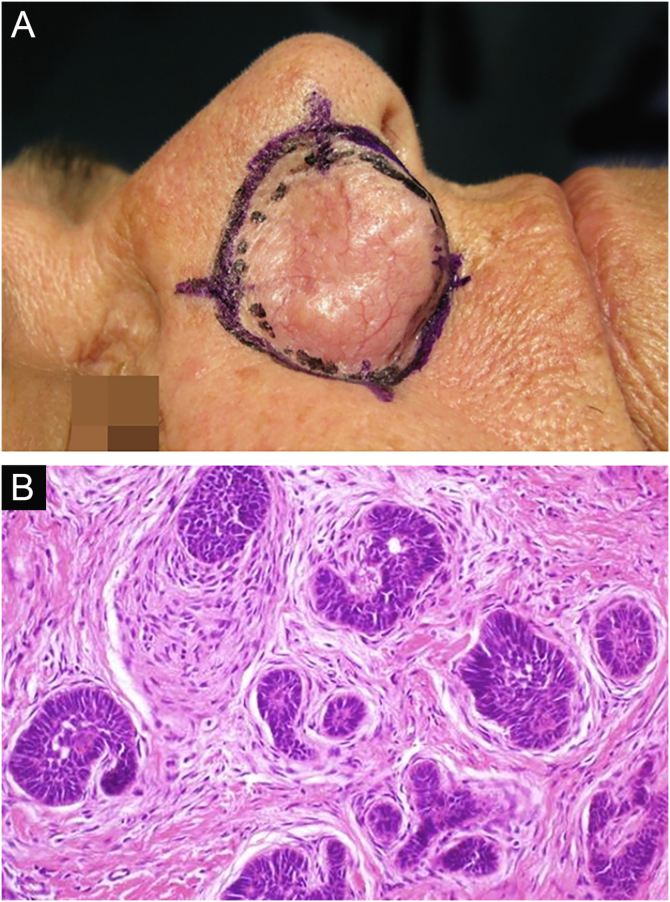
Recurrent trichoblastoma. (A) (top image: clinical delimitation during MMS. (B) (lower image (paraffin-embedded section, Hematoxylin & eosin): proliferation of follicular germ cells forming, islets of basaloid cells inside a fibrocellular stroma. Source: Archives of Dermatology HC-Unicamp.

Among other examples is trichoblastoma, a rare, slow-growing cutaneous adnexal tumor. In this series, there was one case of recurrent trichoblastoma in a 66-year-old female patient (Fig. 3). Negative margins were obtained in two stages, with no signs of recurrence at ten years of follow-up. Although benign, there have been case reports of a more aggressive course, especially in large, recurrent tumors of previous long-term evolution.^30^, ^39^

A case of primary cutaneous adenocarcinoma was also included in this series, diagnosed after multiple paraffin sections and a broad panel of immunohistochemistry, in addition to an extensive and multidisciplinary investigation to exclude a possible primary site other than the skin. There are few reports of adenocarcinoma-like tumors treated by MMS.^35^

**Figure 4:**
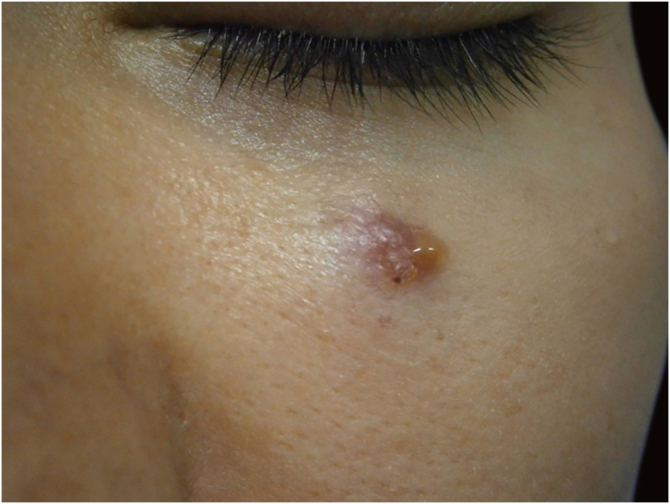
Nodular lesion measuring approximately 1.5 cm below the eyelid with a diagnosis of primary cutaneous adenocarcinoma. Source: Archives of Dermatology HC-Unicamp.

**Figure 5:**
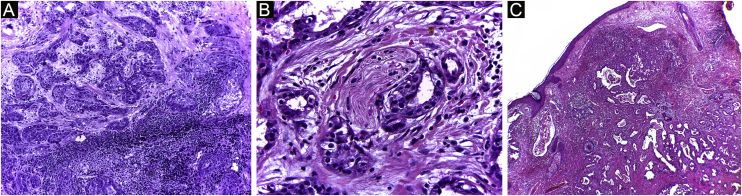
Primary cutaneous adenocarcinoma (Hematoxylin & eosin). (A) (image on the left): 5 micron-thick frozen sections, showing glandular structures infiltrating the reticular dermis, associated with chronic inflammation. (B) (center image): paraffin-embedded section showing neoplastic perineural infiltration. (C) (image on the right): paraffin-embedded section, panoramic view showing a poorly circumscribed tumor, consisting of irregular glandular structures invading the dermis. Source: Archives of Dermatology/Pathological Anatomy HC-Unicamp.

In this peculiar case, the difficulty in the initial definitive diagnosis of the lesion, due to the non-specific clinical characteristics, was associated with alterations in the anatomopathological examination and in the immunohistochemistry analysis, which led to the need to rule out a cutaneous metastasis of a primary tumor elsewhere (Figure 4, Figure 5). After the diagnostic conclusion that it was a primary cutaneous adenocarcinoma, the therapeutic option using Mohs micrographic surgery reinforced the importance of this surgical technique. The patient has been under clinical follow-up for eight years, with no recurrence of the skin lesion and no evidence of another primary site.

It is also noteworthy that the case with the highest number of stages and fragments was a recurrent spiradenoma. Described in 1956 by Kersting and Helwig, spiradenoma is a rare, undifferentiated or poorly differentiated benign neoplasm of the sweat glands. There is a risk of malignant transformation, particularly in older and larger lesions.^38^ The treatment of choice is surgical, but there is no clear recommendation for margins. Little is known about the behavior of these tumors, as they are rare, with few published cases. MMS should be considered in recurrent, large, or critically-located spiradenomas to ensure complete excision and tissue sparing.

**Figure 6:**
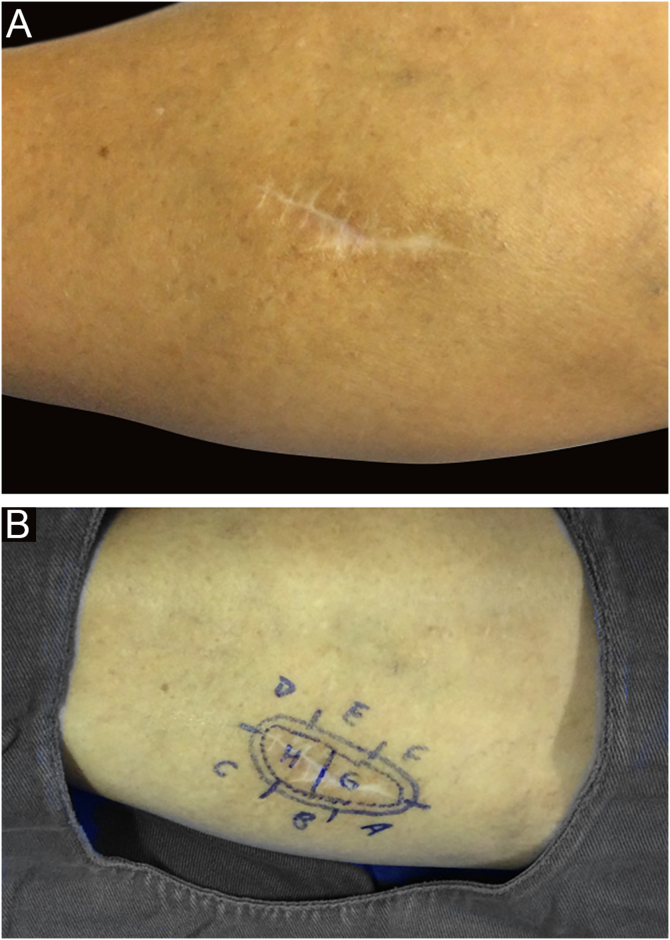
(A) Scar on the right leg secondary to previous conventional surgery. (B) Clinical delimitation of recurrent spiradenoma during MMS. Source: Archives of Dermatology HC-Unicamp.

**Figure 7:**
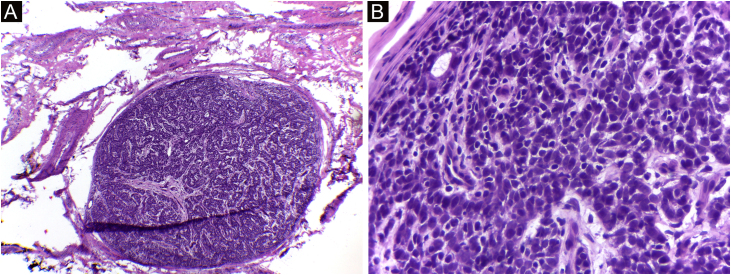
Spiradenoma (5-micron thick frozen sections stained with Hematoxylin & eosin). (A) (image on the left): multinodular pattern, with relatively large and circumscribed nodules within the dermis and subcutaneous tissue. (B) (image on the right): Basaloid cells of two types. In the periphery they are small with hyperchromic nuclei and in the center or around small lumens they are larger with pale nuclei. Source: Archives of Dermatology/Pathological Anatomy HC-Unicamp.

In this case, it was a lesion with compromised margins in the first approach, fast-growing in a few months, and painful. For this reason, MMS was indicated and the fact that it required three stages to obtain negative margins corroborated the indication, demonstrating that the lesion would have been incompletely removed with the conventional surgical technique^33^, ^37^, ^38^ (Figure 6, Figure 7)

**Figure 8:**
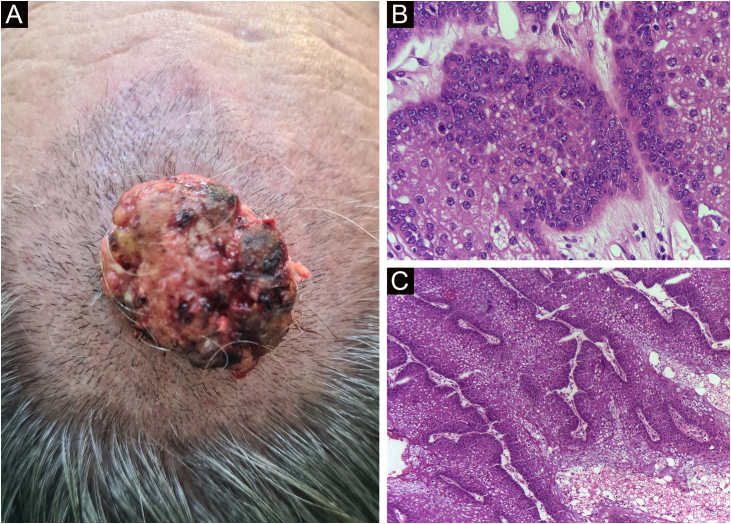
(A) Image on the left: Extraocular sebaceous carcinoma. (B) (Upper right image (paraffin-embedded section): poorly defined lobes of basaloid cells and poorly differentiated sebaceous cells. Moderate atypia. (C) (lower right image 5 micron thick frozen section): tumor debulking during Mohs micrographic surgery. Source: Archives of Dermatology HC-Unicamp.

Finally, the inclusion of a case of sebaceous carcinoma of the scalp (Fig. 8) is highlighted. It is a rare adnexal tumor, and most are located in the periocular region. They are characterized by being fast-growing tumors, often underdiagnosed, with a risk of distant metastasis, in addition to the association with other visceral and cutaneous neoplasms in the Muir-Torre syndrome. A Chinese study analyzed 360 cases located in the eyelids and in only 62.5% of them, sebaceous carcinoma was the initial clinical hypothesis. About 20% included hypotheses of benign lesions such as chalazion, blepharitis, and nevus. MMS was the technique of choice for treatment of 31.9% of these cases and showed better control of local recurrence, with a five-year cure rate of about 90%, when compared to conventional surgery with cure rates varying from 60% to 80%.^40^

Currently, surgical treatment is preferred treatment for all skin tumors, with some indications for non-surgical treatment for superficial and less aggressive lesions, such as superficial BCC, or in patients with contraindications for the procedure.

Regarding rare tumors, due to the absence of well-established treatment criteria and sufficient literature data, the use of non-surgical techniques for initial lesions is rarely indicated. Due to their recurrent characteristic, being incompletely excised by conventional surgery with wide margins, MMS indication is supported by literature data and the expansion of the method should be considered.^5^, ^6^, ^26^, ^27^, ^28^, ^29^, ^30^, ^31^, ^32^, ^33^, ^34^, ^35^, ^36^

## Conclusion

This retrospective case series shows that Mohs micrographic surgery is an appropriate technique for the treatment of rare skin tumors. Regarding the frequency, they corresponded to approximately 5% of all cases treated with MMS during the 12-year period with no recurrences observed after an average of six years of follow-up.

With the benefit of a complete assessment of tumor margins associated with lower rates of recurrence reported in the literature, the possibility of extending the uses of the method should be considered, which could lead to better results.

## Financial support

The present study received support from Funadersp, Fundo de Apoio à Dermatologia de São Paulo - Sebastião Sampaio, project n. 86-2019.

## Authors' contributions

Thais Helena Buffo: Statistical analysis; Approval of the final version of the manuscript; design and planning of the study; drafting and editing of the manuscript; collection, analysis, and interpretation of data; intellectual participation in the propaedeutic and/or therapeutic conduct of the studied cases; critical review of the literature; critical review of the manuscript.

Rafael Fantelli Stelini: Approval of the final version of the manuscript; drafting and editing of the manuscript; collection, analysis, and interpretation of data; effective participation in research orientation; intellectual participation in the propaedeutic and/or therapeutic conduct of the studied cases; critical review of the manuscript.

Juliana Yumi Massuda Serrano: Statistical analysis; approval of the final version of the manuscript; design and planning of the study; intellectual participation in the propaedeutic and/or therapeutic conduct of the studied cases; critical review of the manuscript.

Luciana Takata Pontes: Approval of the final version of the manuscript; design and planning of the study; collection, analysis, and interpretation of data; intellectual participation in the propaedeutic and/or therapeutic conduct of the studied cases; critical review of the manuscript.

Renata Ferreira Magalhães: Approval of the final version of the manuscript; design and planning of the study; effective participation in research orientation; intellectual participation in the propaedeutic and/or therapeutic conduct of the studied cases; critical review of the manuscript.

Aparecida Machado de Moraes: Approval of the final version of the manuscript; design and planning of the study; collection, analysis, and interpretation of data; effective participation in research orientation; intellectual participation in the propaedeutic and/or therapeutic conduct of the studied cases; critical review of the literature; critical review of the manuscript.

## Conflicts of interest

None declared.
